# Filling Teeth

**Published:** 1848-01

**Authors:** E. Taylor


### FILLING TEETH.

BY E. TAYLOR, M. D., D. D. S.

In my former No. I had brought forward my arrangements ready to commence the operation of filling. I will now take up one of the most simple cases to be met with in Dental practice, viz: filling a cavity in the end of a Superior Molar. The first thing generally to be done is to cut away the enamel and thus uncover the cavity, for we usually find that the ravages of disease have been much more extensive in the osseous structure that in the enamel, and we would sometimes find it about as difficult to scrape out the inside of a barrel and fill it up solidly through the bung hole as to properly fill a cavity through the aperture we find in it, although it would be decidedly easier and cheaper too (as some men count cheapness) to fill the bung hole only. The enamel should always be cut away so as to have the opening very nearly if not quite as large as the internal cavity; especially should all the enamel be cut away that is not supported beneath by sound bone. Sometimes a cavity recedes so much in some direction

3

that it is necessary to cut away considerably of the sound bone in the neck of the cavity so as to give an opportunity of filling up under the projection. We also occasionally find but one opening and several chambers to these cavities. It is generally best to form them into one as much as can well be done, as the filling is stronger, having but one body.

For the purpose of cutting away the enamel, I have a pair of well tempered instruments, pointed somewhat like a curved letter *v*, with a sharp point, and the bevel a little greater than is common in sealing instruments. If the edges are kept well sharpened, they cut very expeditiously. Sometimes I use a conical burheaded drill, and indeed generally prefer them in the smaller sized cavities.

After the external opening has been thus prepared, I take an excavator, shaped like the edge of a hatchet and of a suitable size and with a keen edge; with this cut down to the solid bone immediately beneath the enamel, and keeping the edge of the instrument to the solid bone, cut entirely around the neck of the cavity. If the tooth be sensitive, this is usually the most painful part of the operation, and requires to be thoroughly executed. After this, with an instrument hoe shaped with an angle, or curved to have the point set back of a square angle, I proceed to excavate the caries from the bottom of the cavity. In an irregular shaped cavity a greater variety of instruments are needed, suited to the peculiar shape of the cavity, and these being so varied, it would be needless, if not impossible to describe them. An operator's best judgment is constantly in requisition, and he must think and contrive for himself.

To have the carious bone thoroughly eradicated, I regard as the first cardinal principle in the operation, and to have the cavity so shaped as to retain the filling, the second. We might as well expect a filling to remain against a smooth wall as in some cavities shaped as we find them. In cavities, such as I have been describing, we usually find them sufficiently well shaped by the time the caries have been removed; but if they be not, we must proceed by excavators or drills to form at least two antagonistic points so as that they will retain the filling when consolidated within.

This will frequently require much nicety of feeling, or a good mechanical eye or judgment. The process of excavating will be facilitated by keeping the cavity frequently wiped out by a pledget of cotton, and it is frequently well to wash it out with a small syringe.

The cavity being now prepared, the question presents itself, with what shall it be filled? Let us then consider the indications to be met. The tooth is an important organ in the animal economy, useful in its offices and painful in its progress of disease and removal. If the filling should fail it is at least endangered, and if not lost must be refilled, which is unpleasant if not painful to the patient, and expensive either to the patient or operator. It is then evidently my duty to fill it with something that will preserve it for the longest possible period. There are many substances in nature that are durable of themselves, but easily destroyed or decomposed by the action of other substances. The question then arises, to what agencies will the filling be exposed? It is exposed to much pressure; it must therefore be solid or capable of being made so. It is exposed to the fluids of the mouth; it must therefore be impervious and incapable of solution in that fluid. This leads us to examine the character of this fluid, and we would here express our regret that it has not been more carefully examined and submitted to more varied chemical analysis, for it is so varied in its character by the circumstances of its existence that it would require a great many observations to correctly determine its specific character; but experience has shown us that it acts with much power in decomposing animal, vegetable and mineral substances, and that generally it is the nitrous acid that most largely prevails. It should therefore be capable of resisting the chemical action of this fluid. It is also exposed to a considerable amount of heat in the fever heat of the mouth, and by hot drinks and food, and thus requires to be incapable of softening or of being otherwise injuriously affected by a very considerable temperature. The absorbents of the gums and teeth are liable to act upon it, and it is required to resist this agency. It is the duty of the operator to consider all these indications, and to meet them if possible. Well, what substance or substances, simple or compound, will meet them, I shall not attempt to range out of the record. The substances now used I believe by Dentists universally are Gold, Platina, Silver, Tin, Lead and Mercury in componnd with some of the foregoing. Gold we find, when properly prepared, to be soft and capable of being introduced into the cavity, and there consolidated and made firm and capable of resisting any pressure that may be required in its condition. It is, when thus consolidated, impervious to moisture, uninjured by heat, incapable of being absorbed, and only acted upon by the combined action of two powerful acids, which are never found in the mouth in sufficient strength to affect it. It therefore meets all the indications required. But gold is a very expensive metal; can its place be supplied by any less costly material that will serve the purpose as well, and the pockets better. Platina is somewhat less expensive, but has not been used to any extent for this purpose. The foil that I have seen of it is too hard, and wanting in adhesiveness; but from the softness and malability of the wire I should think it capable of being made useful, but at present it is hardly subject to consideration. The same objections lie against silver, with the additional fact that it is so readily acted upon by the acids of the buccal cavity, that its use, which was never considerable, has been now almost entirely abandoned. Tin has more of the qualities desired than either of the metals last mentioned, but it is liable to be acted upon by the acids and oxidized, and thus becomes dark, and is therefore particularly unfit for the front teeth or cavities that are conspicuous; but of all the cheaper material I consider it by far the best, and regard its use sometimes fully justifiable, particularly in deciduous teeth and in very large cavities, as it is not every one that can afford the expense of "nine leaves of gold foil and four hours labor" by the Dentist on a single tooth. But where the patient can afford it, and the tooth can be permanently saved, gold should always be used.

Lead is more easily acted upon by acids and is oxidized by the atmosphere, and has nothing to commend its use.

Mercury, combined with some other metal forming an amalgam, has the advantage of being soft and plastic, is more readily introduced, and compressed to fill the cavity, and soon hardens and becomes very firm. It is sometimes amalgamated with gold, but generally with silver, which is fully as good, as "a chain can never be stronger than its weakest link."

The objections to its use are, that it is easily acted upon by the acids of the mouth, and may in its new relation be highly prejudicial. The combination of two metals promotes galvanic action, and is very injurious to the organism of the teeth. It may also be acted upon by the absorbents of the gums and teeth, and thus in its minute divisions has its specific action, upon numerous well attested cases of Ptyalism, been fully established. I have a tooth now in my possession that had been filled with the amalgam for a few weeks, for a young miss of about fourteen years of age. The whole osseous structure of the tooth was colored to a green almost equal to a forest leaf, and became so excruciatingly painful as to require extraction. How it produced this peculiar discoloration I am not prepared to say, but suppose it must have been effected through the action of the absorbent vessels of the tooth.

We are then driven to the conclusion that gold is the only material yet known that meets all the indications desired in filling a tooth. But even this is much varied in its quality, and I would just as soon expect to fill a tooth permanently with corn husks as with some of the foil we sometimes meet with, as it appears entirely wanting in body and adhesiveness, while some again is so hard that it is incapable of being used. The qualities most desired in gold foil are softness and adhesiveness. We suppose these depend upon the purity of the gold and the manner of working it, and no manufacturer that we have met with makes it uniformly alike in its quality.

We use No. 6 more than any other No., yet for large cavities we prefer 8 or even 12; but for these heavier Nos. softness is indispensable.

I prepare my foil by folding it to four or six thicknesses, and use one or more leaves together as the size of the cavity may require. Then cut it in strips or a strip from a sixteenth to a thirtieth of an inch in width, corresponding to the size of the cavity. I do not cut the strips entirely off, but reverse the ends alternately, so that my foil is all in one strip.* This I consider an important advantage, as it is frequently troublesome and tedious to start on a new strip.

But it is too late to insert the filling this evening, so it must be postponed until another day.

